# The effect of perceived supportiveness of instruction on high school students' sport ethics orientation: the mediating role of sport learning motivation

**DOI:** 10.3389/fpsyg.2026.1790018

**Published:** 2026-06-05

**Authors:** Yuanhong Qiu, Xin Zhang

**Affiliations:** School of Physical Education, Southwest University, Chongqing, China

**Keywords:** high school students, need-supported teaching perception, physical education learning motivation, self-determination theory, sport ethics orientation

## Abstract

**Background:**

Against the backdrop of “cultivating virtue and nurturing talent” becoming the fundamental mission of education in the new era, physical education courses leverage their inherent practicality, rule-based nature, and collaborative characteristics. Serving as a key vehicle for integrating moral education and helping students develop well-rounded personalities, this approach aligns with the national policy of “cultivating character through physical education” and is an essential requirement of the “five-education” system. However, in current high school physical education instruction, frequent instances of unethical conduct and insufficient student motivation have become prominent issues, directly hindering the effective fulfillment of physical education's role in nurturing students.

**Aim:**

This study examines the relationship among high school students' perceptions of needs-based instruction, their motivation for physical education, and their ethical attitudes toward physical education, as well as the mediating role of motivation for physical education.

**Methods:**

The study sample consisted of 525 students from three high schools in Chongqing, China. The researchers administered the Physical Education Perceived Supportiveness Scale, the Physical Education Learning Motivation Scale, and the Multidimensional Ethical Orientation in Physical Education Scale.

**Results:**

Perceived need-supportive teaching, physical education learning motivation, and ethical orientation in physical education exhibited significant positive correlations (*r* = 0.304, *r* = 0.559, *r* = 0.382, *p* < 0.01). Physical education learning motivation partially mediated the relationship between perceived need-supportive teaching and ethical orientation in physical education (mediation effect accounting for 56.6%).

**Conclusions:**

Perceived need-supportive instruction positively predicts students' motivation to learn physical education and their ethical orientation. Higher levels of motivation to learn physical education further positively predict ethical orientation and partially mediate the effect of need-supportive instruction on ethical development. These findings indicate that a need-supportive instructional environment is crucial for fostering students' intrinsic motivation to learn physical education and cultivating ethical qualities in physical education.

## 1 Introduction

Physical education with a focus on moral integrity serves as a vital pathway for fulfilling the fundamental mission of fostering virtue through education, holding critical significance within China's contemporary high school education system. At the policy level, the “Opinions on Comprehensively Strengthening and Improving School Physical Education in the New Era” explicitly establishes “fostering virtue through education” as the core objective of school physical education, emphasizing the need to “shape sound character and temper strong will” through physical exercise (General Office of the Central Committee of the Communist Party of China and General Office of the State Council, (2020); Zhou and Cheng, 2024; Ma, 2020; Mao et al., 2023). This requirement underscores the state's strategic positioning of physical education as a vital component of holistic education, reflecting its role in the “five-pronged approach to education” (Ministry of Education of the People's Republic of China, 2015, 2026; General Office of the Central Committee of the Communist Party of China and General Office of the State Council, (2015); Zha and Liang, 2024). Unique Value Within the System—Its inherent practicality, rule-based nature, and collaborative characteristics make it a natural and high-quality setting for moral education.

However, the reality is that the physical education standards of high school students face severe challenges: According to a 2022 survey by the Ministry of Education, as many as 65% of high school students engage in less than 3 h of physical activity per week—a 28% decrease from their junior high school years. This has resulted in the policy-mandated “moral education integration” losing its essential practical foundation (Ji, 2022). Meanwhile, the pressure from physical education assessments has led 63.5% of PE teachers to admit that they “overemphasize skill performance at the expense of moral education.” Compounded by the influence of online sports subcultures, some students exhibit blurred perceptions of sports ethics, unruly behavior, and a lack of motivation in learning (Zhao et al., 2020; Manoli et al., 2024; Qian et al., 2024). Against this backdrop, exploring effective teaching approaches to enhance students' sports ethics has become an urgent issue that requires resolution.

Traditional, teacher-centered, control-oriented teaching models often struggle to inspire students' intrinsic identification, potentially leading to passive compliance or even resistance toward sports regulations and ethical requirements (Hu, 2022; Coterón et al., 2024; Leyton-Román et al., 2021). Against this backdrop, needs-supportive teaching grounded in self-determination theory demonstrates tremendous potential for application (Deci and Ryan, 1985). This teaching paradigm emphasizes that educators meet students' fundamental psychological needs by providing autonomy support (empowering choice), competence support (effective feedback), and emotional support (expressing care), thereby fostering the development of intrinsic motivation and positive behaviors (Deci and Ryan, 2000; Zhang et al., 2024; Kruse et al., 2024). Extensive research confirms that supportive teaching significantly enhances students' learning engagement and physical and mental well-being (Cheon et al., 2018; Standage et al., 2005).

Physical education (PE) is increasingly recognized as a means of improving physical fitness and as a crucial platform for moral and character development (General Office of the Central Committee of the Communist Party of China and General Office of the State Council, (2020); Jacobs et al., 2013). In contemporary education, particularly under the philosophy of fostering virtue through education (“cultivating character through sports”, for example), developing students' positive ethical attitudes toward sport—such as fair play, respect for the rules, and teamwork—has become a key educational objective (General Office of the Central Committee of the Communist Party of China and General Office of the State Council, (2020); Liu et al., 2023; General Administration of Sport of China Ministry of Education, 2020). However, identifying the mechanisms that effectively promote these behaviors in daily PE practice remains a significant challenge. Existing research has separately explored perceptions of demand-supportive teaching, physical education learning motivation, and ethical orientations in physical education (Maldonado et al., 2019; Guo et al., 2025; Hui et al., 2022). Internationally, self-determination theory (SDT) has been widely applied in educational motivation studies, indicating that the influence of external environments on individual behavior often requires the activation of intrinsic motivation to be realized (Ryan and Deci, 2020; Wang Y. et al., 2024a; Wang et al., 2024b). Meta-analytic evidence from 36 SDT-based interventions further demonstrates that need-supportive educational environments significantly enhance students‘ intrinsic motivation and basic psychological need satisfaction (Wang Y. et al., 2024a; Wang et al., 2024b). In the domain of physical education, a systematic review found that SDT-informed teaching strategies effectively foster autonomy-supportive environments and enhance student motivation (Zhou et al., 2025), while empirical research confirms that need-supportive social environments positively influence intrinsic motivation through the mediation of basic psychological needs (Chu et al., 2019). Domestic research has primarily focused on single-variable relationships, such as the impact of teacher support on student motivation (Hu, 2022; Sun and Ji, 2010; Zhang et al., 2018; Zhang, 2012). Although some studies indicate a positive correlation between autonomy-supportive teaching and sports ethical orientation (Fang, 2023), with sports learning motivation potentially mediating this relationship—when students engage in physical activities driven by interest and enjoyment (intrinsic motivation), they are more likely to internalize sportsmanship and exhibit positive ethical behaviors such as respecting rules and fair competition (Hodge and Lonsdale, 2011)—research integrating these three elements into a systematic model remains scarce, particularly lacking empirical validation among Chinese high school students. This research gap constrains the effective implementation of the “cultivating character through sports” philosophy in daily teaching practices.

Therefore, based on self-determination theory, this study aims to explore the mechanism through which perceived need-supportive teaching influences Chinese high school students' ethical orientation in physical education, as well as the mediating role of learning motivation in physical education. Specifically, the following hypotheses are proposed:

H1: Perceived need-supportive teaching by physical education teachers positively predicts high school students' ethical orientation in physical education;

H2: Perceived need-supportive teaching by physical education teachers positively predicts high school students' learning motivation in physical education;

H3: High school students' learning motivation in physical education positively predicts their ethical orientation in physical education;

H4: High school students' physical education learning motivation mediates the effect of perceived demand-supportive teaching by physical education teachers on their ethical orientation in physical education.

## 2 Materials and methods

### 2.1 Ethical considerations

This study was approved by the Ethics Review Com-mittee of Southwest University, China (Approval Code: SWU-PE-20250311; Approval Date: March 11, 2025), adhering to une Decraration of Helsinki. Participants were informed of procedures, risks, benefits, confidentiality, and withdrawal rights via Mandarin Chinese interviews. Written informed consent was obtained, ensuring volun-tary participation and compliance with ethical standards. No compensation was provided. Data were de-identified, with personal information securely stored and accessible only to researchers.

### 2.2 Participants

Using random sampling, 560 high school students from Southwest University Affiliated High School, Chaoyang High School, and No. 29 High School in Chongqing, China, were selected as survey subjects. A total of 544 questionnaires were successfully collected, achieving a response rate of 97.1%. During questionnaire review, those with incomplete responses, identical answers, or obviously abnormal responses were deemed invalid and excluded. A total of 19 questionnaires were discarded, resulting in 525 valid responses (Table 1). The final valid response rate was 96.5%.

**Table 1:** Distribution of demographic variables among survey respondents (*N* = 525).

Variable	Category	Number of people	Percentage (%)
Gender	Male	270	51.4
	Female	255	48.6
Grade	Freshman	205	39.0
	Sophomore	172	32.8
	Senior	148	28.2
Are you an only child?	Yes	251	47.8
	No	274	52.2

The survey was conducted with the authorization of the principal, physical education teachers, and students. Paper questionnaires were distributed to students by class. Before distributing the questionnaires, teachers explained their purpose and instructions to the students. All questionnaires were administered uniformly during the PE class. Participants were asked to complete the questionnaire independently within 10 min. Teachers supervised the entire process to ensure students answered truthfully and to prevent discussion among students, and collected the completed questionnaires immediately afterward. After manually entering the data, SPSS 27.0 and the PROCESS plugin were used for data analysis.

### 2.3 Data collection tools

This study utilized a questionnaire consisting of four sections: an informed consent form, the Perceived Supportive Teaching in Physical Education Scale, the Physical Education Learning Motivation Scale, and the Multidimensional Ethical Orientation in Physical Education Scale.

Using Hu Xiaoqing's revised 〈〈Perception Scale for Support-Oriented Teaching in Physical Education Classes〉〉 (Hu, 2022). This scale comprises 11 items across three dimensions: autonomy support, competence support, and emotional support. It employs a 7-point Likert scale, where “strongly disagree” scores 1 point and “strongly agree” scores 7 points. Cronbach's α is 0.928, and the structural validity test (KMO value) is 0.911, both indicating excellent reliability and validity for this instrument.

Using Hu Xiaoqing's revised 〈〈Physical Education Learning Motivation Scale〉〉 (Hu, 2022). This scale comprises 15 items across five dimensions: amotivation, external regulation, introspection, identification, and intrinsic motivation. It employs a 7-point Likert scale, where “strongly disagree” scores 1 and “strongly agree” scores 7. The calculation formula is: Internal Regulation × 2 + Identification Regulation – Introversion Regulation – External Regulation × 2 – Amotivation × 3. Cronbach's α is 0.827, and the structural validity test (KMO value) is 0.825, both indicating excellent reliability and validity of this instrument.

Using Sun Kaihong's revised 〈〈Multidimensional Sport Ethics Orientation Scale〉〉 (Sun, 2011). The scale comprises 17 items across four dimensions: social norms, rule adjudication, respect for opponents, and instrumental aggression. It employs a Likert 5-point scale, where “strongly disagree” scores 1 and “strongly agree” scores 5. When calculating the mean for sports ethics orientation, the “instrumental aggression” dimension requires reverse scoring. Cronbach's α was 0.861, and the structural validity test (KMO value) was 0.864, both indicating excellent reliability and validity of this instrument.

### 2.4 Validity and reliability testing

Conducting reliability and validity analyses of the questionnaire is a critical method for evaluating its measurement quality. In this study, reliability and validity testing of the survey questionnaire will strictly adhere to statistical standards (Jiang et al., 2010) (Tables 2, 3).

**Table 2:** Reference standard for Cronbach's alpha coefficient.

Kronbach's alpha coefficient range	Internal consistency
α ≥ 0.9	Highly reliable
0.9 > α ≥ 0.8	Generally
0.8 > α ≥ 0.7	Good
0.7 > α ≥ 0.6	Barely acceptable
0.6 > α	Unacceptable

**Table 3:** KMO Coefficient and Bartlett's test of sphericity reference standards.

Statistic	Numerical range	Interpretation
KMO coefficient	KMO ≥ 0.9	Excellent validity
	0.9 > KMO ≥ 0.8	Average
	0.8 > KMO ≥ 0.7	Good
	0.7 > KMO ≥ 0.6	Barely acceptable
	0.6 > KMO	Unacceptable
Bartlett's sphericity test (P)	0.001 > P	Strongly significant
	0.05 > P	Significant
	P ≥ 0.05	Not significant

#### 2.4.1 〈〈Perception Scale for Support-Oriented Teaching in Physical Education Classes〉〉

##### 2.4.1.1 Reliability test

All three subscales in this study demonstrated high levels of reliability. Specifically, the Cronbach's α coefficient for the Autonomous Support subscale was 0.861; for the Competence Support subscale, it was 0.883; and for the Emotional Support subscale, it was 0.822. The Cronbach's α coefficient for the total scale was 0.919, further confirming the overall reliability of the scale (Table 4).

**Table 4:** Reliability test of the perceived support teaching scale for physical education class.

Subscale	Number of items	Klonbach alpha
Self-support	4	0.861
Competency support	4	0.883
Emotional support	3	0.822
Demand-supported teaching perception	11	0.919

##### 2.4.1.2 Validity test

According to the data presented in Table 5, the KMO value is 0.923, and Bartlett's sphericity test yields a significant result (X^2^ = 3,394.563, df = 55, *P* < 0.001). These findings collectively indicate that the scale possesses good construct validity.

**Table 5:** KMO values and Bartlett's test.

KMO coefficient	Parameter	0.923
Bartlett's sphericity test	*X^2^*	3,394.563
	Degrees of freedom	55
	Significance level	0.000

#### 2.4.2 “Physical Education Learning Motivation Scale”

##### 2.4.2.1 Reliability test

All five subscales involved in this study demonstrated high levels of reliability. Specifically, the Cronbach's α coefficient for the Amotivational Scale was 0.882; for the External Regulation Scale, it was 0.891; the internal regulation subscale had a Cronbach's α coefficient of 0.889; the identity regulation subscale had a Cronbach's α coefficient of 0.859; and the internal motivation subscale had a Cronbach's α coefficient of 0.886. The total scale achieved a Cronbach's α coefficient of 0.956, further confirming the overall reliability of the scale (Table 6).

**Table 6:** Reliability test of the physical education learning motivation scale.

Subscale	Number of items	Clonbach alpha
Unmotivated	4	0.882
External regulation	3	0.891
Intrinsic regulation	2	0.889
Identification regulation	3	0.859
Internal motivation	3	0.886
Motivation for physical education learning	15	0.956

##### 2.4.2.2 Validity test

According to the data presented in Table 7, the KMO value is 0.952, and Bartlett's sphericity test yields a significant result (X^2^ = 6,527.355, df = 105, *P* < 0.001). These findings collectively indicate that the scale possesses good construct validity.

**Table 7:** KMO values and bartlett's test.

KMO coefficient	Parameter	0.952
Bartlett's sphericity test	*X^2^*	6,527.355
	Degrees of freedom	105
	Significance level	0.000

#### 2.4.3 “Multidimensional Sport Ethics Orientation Scale”

##### 2.4.3.1 Reliability test

All five subscales involved in this study demonstrated high levels of reliability. Specifically, the Cronbach's α coefficient for Social Norms was 0.885; for Rule Enforcement, it was 0.856; for Respect for Opponents, it was 0.885; and instrumental aggression at 0.955. The total scale achieved a Cronbach's α coefficient of 0.957, further validating the overall reliability of the scale (Table 8).

**Table 8:** Reliability test of the multidimensional sports ethics orientation scale.

Subscale	Number of items	Clonbach alpha
Social norms	5	0.885
Rule enforcement	4	0.856
Respect for opponents	4	0.885
Instrumental aggression	4	0.960
Ethical orientation in sports	17	0.957

##### 2.4.3.2 Validity test

According to the data presented in Table 9, the KMO value is 0.951, and Bartlett's sphericity test yields a significant result (X^2^ = 7,768.885, df = 136, *P* < 0.001). These findings collectively indicate that the scale possesses good construct validity.

**Table 9:** KMO values and Bartlett's test.

KMO coefficient	Parameter	0.951
Bartlett's sphericity test	*X^2^*	7,768.885
	Degrees of freedom	136
	Significance level	0.000

## 3 Research results

### 3.1 Common method bias test

This study employed the self-report questionnaire as the sole data collection tool, a method that may introduce common method bias. To address this potential bias, the study systematically tested for common method bias in the data using Harman's single-factor test, following the recommendations of scholars such as Zhou Hao and Long Lirong (Zhou and Long, 2004). The analysis results indicate that the eigenvalues of all six factors exceed 1. The variance explained by the first principal factor is 36.56%, which falls below the 40% threshold. This suggests that no significant homogeneity error exists in this study, allowing for the continuation of subsequent research (Table 10).

**Table 10:** Total variance explained.

Ingredients	Initial eigenvalues			Extracted load squares		
	Total	Percentage of variance	Cumulative %	Total	Percentage of variance	Cumulative %
1	15.721	36.56	36.56	15.721	36.56	36.56
2	6.745	15.687	52.247	6.745	15.687	52.247
3	3.379	7.859	60.106	3.379	7.859	60.106
4	1.301	3.026	63.132	1.301	3.026	63.132
5	1.207	2.806	65.938	1.207	2.806	65.938
6	1.168	2.716	68.654	1.168	2.716	68.654
7	0.973	2.263	70.917			
8	0.775	1.802	72.719			
……	……	……	……	……	……	……

Due to the excessive number of items, only the sections relevant to the reported values are included.

### 3.2 Descriptive statistics and tests of differences for key variables

This study employed descriptive statistical analysis in SPSS 27.0 to describe each variable and its dimensions. Independent samples *t*-tests were conducted to examine differences in perceptions of needs-supportive teaching, physical education learning motivation, and ethical orientation in physical education, with high school students‘ gender and whether they were only children serving as grouping variables. Additionally, one-way ANOVA tests were performed to examine differences in perceptions of needs-supportive teaching, physical education learning motivation, and ethical orientation in physical education, with high school students' grade level as the factor.

#### 3.2.1 Demand-supported teaching perception

##### 3.2.1.1 Descriptive statistics

The highest perceived score for demand-driven teaching was 7, the lowest was 1, with an average score of 6.126 ± 0.665 (on a scale of 1–7). The distribution of respondents across each score range is shown: 1 point (0.21%), 2 points (0.33%), 3 points (0.92%), 4 points (2.01%), 5 points (16.68%), 6 points (41.47%), and 7 points (38.39%). Based on the average score and the distribution of students across score bands, high school students' perceived demand support is generally at an upper-middle level (Table 11).

**Table 11:** Descriptive statistics of perceived demand-supported teaching.

Category	Sample size	Mean	Standard deviation	Minimum	Maximum
Need-supported teaching perception	525	6.126	0.665	1	7
Autonomy support	525	6.078	0.758	1	7
Competence support	525	6.096	0.778	1	7
Emotional support	525	6.229	0.743	1	7

##### 3.2.1.2 Differential analysis

① Differential analysis of perceived dimensions in needs-supportive teaching based on whether students are only children.

High school students' perceptions of need-supportive teaching (*t* = 0.638, *P* = 0.524), autonomy-supportive teaching (*t* = 1.054, *P* = 0.292), competence-supportive teaching (*t* =−0.016, *P* = 0.987), and affective support (*t* = 0.682, *P* = 0.496) showed no significant differences based on whether they were only children (Table 12).

**Table 12:** Differences in perceived dimensions of needs-supportive teaching between only children and non-only children.

Category	Gender	Number of cases	Mean	Standard deviation	*t*	*P*
Needs-supported teaching perception	Yes	251	6.145	0.59	0.638	0.524
Autonomy support	No	274	6.108	0.722		
Competence support	Yes	251	6.115	0.710	1.054	0.292
Emotional support	No	274	6.045	0.799		
Category	Yes	251	6.096	0.718	−0.016	0.987
Needs-supported teaching perception	No	274	6.097	0.831		
Autonomy support	Yes	251	6.252	0.716	0.682	0.496
	No	274	6.208	0.769		

② Analysis of grade-level differences in perceived dimensions of needs-supported teaching.

High school students‘ perceptions of need-supportive teaching (*F* = 9.351, *P* = 0.000), autonomy support (*F* = 4.821, *P* = 0.008), and competence support (*F* = 6.363, *P* = 0.002) and affective support (*F* = 12.923, *P* = 0.000) across all grade levels. Scoring patterns revealed that lower-grade students (10th graders) perceived significantly lower levels of need support compared to upper-grade students, while 11th graders reported the highest perceived scores across all support dimensions. This phenomenon can be explained by students' varying stages of adaptation and academic pressure: Freshmen, newly transitioned to high school, require adjustment to their new environment and mindset, resulting in the lowest perceived need support. Sophomores, having fully adapted and experiencing relatively balanced academic pressure, exhibit the highest perceived scores. Juniors, though familiar with teachers, face college entrance exam pressure that diverts psychological resources toward academics, suppressing their perception of non-academic support and yielding lower scores than sophomores (Table 13).

**Table 13:** Test for differences in dimensions of perceived demand-support teaching across grades.

Category	Gender	Number of cases	Mean	Standard deviation	*F*	*P*
Need-supported teaching perception	Freshman	205	5.979	0.7	9.351	0.000^**^
	Sophomore	172	6.265	0.519		
	Senior	148	6.167	0.693		
Autonomy support	Freshman	205	5.952	0.819	4.821	0.008^**^
	Sophomore	172	6.179	0.649		
	Senior	148	6.135	0.768		
Competence support	Freshman	205	5.951	0.823	6.363	0.002^**^
	Sophomore	172	6.225	0.678		
	Senior	148	6.147	0.796		
Emotional support	Freshman	205	6.052	0.754	12.923	0.000^**^
	Sophomore	172	6.434	0.602		
	Senior	148	6.237	0.816		

^*^P < 0.05.

^**^P < 0.01.

#### 3.2.2 Motivation for physical education learning

##### 3.2.2.1 Descriptive statistics

The highest score for physical education learning motivation was 15, the lowest was −37.17, and the average score was 6.211 ± 8.247 (Physical Education Learning Motivation = Internal Regulation × 2 + Identified Regulation – Introjected Regulation – Extrinsic Regulation × 2 – Amotivation × 3) (Table 14). The distribution of participants across score bands was as follows: 1 point (0.47%), 2 points (0.83%), 3 points (2.18%), 4 points (8.81%), 5 points (16.79%), 6 points (25.54%), and 7 points (45.38%). Based on the average scores and the distribution of students across different score ranges, high school students' learning motivation shows an overall positive trend.

**Table 14:** Descriptive statistics of sports learning motivation.

Name	Sample size	Mean	Standard deviation	Minimum	Maximum
Sports learning motivation	525	6.211	8.247	−37.17	15
Unmotivated	525	1.796	0.935	1	7
External regulation	525	1.965	1.032	1	7
Internal regulation	525	2.047	1.190	1	7
Identification regulation	525	5.750	1.111	1	7
Intrinsic motivation	525	5.912	1.092	1	7

##### 3.2.2.2 Differential analysis

① Differential Analysis of Dimensions of Sports Learning Motivation Based on Whether Students Are Only Children.

High School Students' Motivation for Physical Education Learning (*t* = 0.012, *P* = 0.990), without motivation (*t* = 0.488, *P* = 0.626), External Regulation (*t* =−0.177, *P* = 0.859), Internal focus adjustment (*t* = 0.970, *P* = 0.332), Identity Regulation (*t* =−0.698, *P* = 0.486), and intrinsic motivation (*t* =−0.587, *P* = 0.557) showed no significant differences based on whether students were only children (Table 15).

**Table 15:** Test of differences in various dimensions of sports learning motivation between only children and non-only children.

Category	Gender	Number of cases	Mean	Standard deviation	*t*	*P*
Motivation for physical education learning	Yes	251	6.215	7.927	0.012	0.990
	No	274	6.207	8.544		
Without motivation	Yes	251	6.225	0.90	0.488	0.626
	No	274	6.185	0.964		
External regulation	Yes	251	6.027	1.00	−0.177	0.859
	No	274	6.043	1.058		
Internal exposure adjustment	Yes	251	6.006	1.08	0.970	0.332
	No	274	5.905	1.275		
Identity regulation	Yes	251	5.715	1.07	−0.698	0.486
	No	274	5.782	1.141		
Intrinsic motivation	Yes	251	5.883	1.08	−0.587	0.557
	No	274	5.939	1.095		

② Analysis of Grade-Level Differences in Dimensions of Sports Learning Motivation.

High school students‘ motivation for physical education learning (*F* = 32.284, *P* = 0.000), lack of motivation (*F* = 26.540, *P* = 0.000), external regulation (*F* = 13.796, *P* = 0.000), internal regulation (*F* = 8.154, *P* = 0.000), identity regulation (*F* = 40.822, *P* = 0.000), and intrinsic motivation (*t* = 41.988, *P* = 0.000) showed significant differences across grade levels. Scoring patterns revealed that lower-grade students (10th graders) exhibited significantly lower physical education learning motivation than upper-grade students, while 11th graders achieved the highest motivation scores across all supporting dimensions. This phenomenon can be explained by students' varying stages of adaptation and academic pressure: Freshmen, newly enrolled in high school, require time to adjust to the new environment and their mindset, resulting in the lowest physical education learning motivation scores. Sophomores have fully adapted to the environment and experience relatively balanced academic pressure, leading to the highest motivation scores. Juniors, though familiar with teachers, face intense college entrance exam pressure that diverts psychological resources toward academics, suppressing non-academic motivational support and resulting in lower scores than sophomores (Table 16).

**Table 16:** Test of differences in various dimensions of sports learning motivation across grade levels.

Category	Gender	Number of cases	Mean	Standard deviation	*F*	*P*
Motivation for physical education learning	Freshman year	205	2.934	8.361	32.284	0.000^**^
	Sophomore year	172	9.225	7.133		
	Senior year	148	7.246	7.718		
Without motivation	Freshman year	205	5.857	1.022	26.540	0.000^**^
	Sophomore year	172	6.496	0.720		
	Senior year	148	6.346	0.881		
External regulation	Freshman year	205	5.763	1.031	13.796	0.000^**^
	Sophomore year	172	6.300	0.991		
	Senior year	148	6.104	0.994		
Internal exposure adjustment	Freshman year	205	5.705	1.198	8.154	0.000^**^
	Sophomore year	172	6.180	1.227		
	Senior year	148	6.034	1.072		
Identity regulation	Freshman year	205	5.283	1.097	40.822	0.000^**^
	Sophomore year	172	6.244	0.914		
	Senior year	148	5.822	1.079		
Intrinsic motivation	Freshman year	205	5.424	1.150	41.988	0.000^**^
	Sophomore year	172	6.357	0.866		
	Senior year	148	6.072	0.976		

^*^P < 0.05.

^**^P < 0.01.

#### 3.2.3 Ethical orientation in sports

##### 3.2.3.1 Descriptive statistics

As shown in Table 17, the highest score for sports ethics orientation was 5, the lowest was 1, and the average score was 4.329 ± 0.718 (on a scale of 1–5). The distribution of participants across score bands is illustrated: 1 point (1.43%), 2 points (3.70%), 3 points (14.36%), 4 points (21.52%), and 5 points (58.98%). Based on the average score and the distribution across score bands, the ethical orientation of high school students overall exhibits a positive trend. The average scores for the four dimensions of sports ethics orientation were as follows: Social Norms 4.305 ± 0.797, Rules and Refereeing 4.384 ± 0.722, Respect for Opponents 4.135 ± 0.875, and Instrumental Aggression 1.501 ± 0.838.

**Table 17:** Descriptive statistics of sports ethics orientation.

Name	Sample size	Mean	Standard deviation	Minimum	Maximum
Sportsmanship orientation	525	4.329	0.718	1	5
Social norms	525	4.305	0.797	1	5
Rule enforcement	525	4.384	0.722	1	5
Respect for opponents	525	4.135	0.875	1	5
Instrumental aggression	525	1.501	0.838	1	5

##### 3.2.3.2 Differential analysis

① Differential analysis of dimensions of sport ethics orientation based on whether participants are only children.

High school students' sports ethics orientation (*t* = 0.267, *P* = 0.790), social norms (*t* = 0.625, *P* = 0.532), rule-based adjudication (*t* = 0.822, *P* = 0.412), respect for opponents (*t* =−1.043, *P* = 0.297), and instrumental aggression (*t* = 0.611, *P* = 0.542) showed no significant differences based on whether participants were only children (Table 18).

**Table 18:** Test of differences in sports ethics orientation dimensions based on only-child status.

Category	Gender	Number of cases	Mean	Standard deviation	*t*	*P*
Sportsmanship orientation	Yes	251	4.338	0.714	0.267	0.790
Social norms	No	274	4.321	0.723		
Rule enforcement	Yes	251	4.328	0.799	0.625	0.532
Respect for opponents	No	274	4.284	0.796		
Instrumental aggression	Yes	251	4.411	0.725	0.822	0.412
Sportsmanship orientation	No	274	4.360	0.720		
Social norms	Yes	251	4.094	0.912	−1.043	0.297
Rule enforcement	No	274	4.173	0.839		
Respect for opponents	Yes	251	4.522	0.830	0.611	0.542
	No	274	4.477	0.846		

② Analysis of grade-level differences across dimensions of sport ethics orientation.

High school students‘ sports ethics orientation (*F* = 16.392, *P* = 0.000), social norms (*F* = 20.305, *P* = 0.000), rules and refereeing (*F* = 12.799, *P* = 0.000), respect for opponents (*F* = 18.694, *P* = 0.000), instrumental aggression (*F* = 3.467, *P* = 0.032) showed significant differences across grade levels (Table 19). The score differences specifically manifest as lower-grade students (10th graders) exhibiting significantly lower sports ethics orientation than upper-grade students, while 11th graders achieved the highest ethical scores across all support dimensions. This phenomenon can be explained by considering students' varying stages of adaptation and academic pressure: Freshmen, having just entered high school, need to adjust to a new environment and mindset, resulting in the lowest ethical orientation scores. Sophomores have fully adapted to their environment and experience relatively balanced academic pressure, leading to the highest ethical orientation scores. Juniors, while familiar with teachers, face intense college entrance exam pressure that diverts psychological resources toward academics, suppressing their intrinsic pursuit of ethical sports behavior and resulting in scores lower than sophomores.

**Table 19:** Test of differences in various dimensions of sports ethics orientation across grade levels.

Category	Gender	Number of cases	Mean	Standard deviation	*F*	*P*
Sportsmanship orientation	Freshman	205	4.142	0.755	16.392	0.000^**^
Social norms	Sophomore	172	4.555	0.607		
Rule enforcement	Senior	148	4.326	0.716		
Respect for opponents	Freshman	205	4.062	0.847	20.305	0.000^**^
Instrumental aggression	Sophomore	172	4.567	0.658		
Sportsmanship orientation	Senior	148	4.335	0.777		
Social norms	Freshman	205	4.220	0.771	12.799	0.000^**^
Rule enforcement	Sophomore	172	4.589	0.587		
Respect for opponents	Senior	148	4.375	0.738		
Instrumental aggression	Freshman	205	3.895	0.916	18.694	0.000^**^
Sportsmanship orientation	Sophomore	172	4.430	0.720		
Social norms	Senior	148	4.125	0.882		
Rule enforcement	Freshman	205	4.411	0.935	3.467	0.032^*^
	Sophomore	172	4.632	0.701		
	Senior	148	4.465	0.826		

^*^P < 0.05.

^**^P < 0.01.

### 3.3 Correlation analysis of perceived need-supported instruction, physical education learning motivation, and ethical orientation in physical education

#### 3.3.1 Demand-supported teaching perception and sport ethics orientation

The total perceived score of demand-support teaching among high school students showed significant positive correlations with three factors under the sports ethics orientation dimension: social norms (*r* = 0.298, *P* < 0.01), rule-based refereeing (*r* = 0.268, *P* < 0.01), and respect for opponents (*r* = 0.287, *P* < 0.01). Conversely, the total perceived score showed a significant negative correlation with instrumental aggression (*r* = −0.222, *P* < 0.01). Furthermore, the total perceived score for demand-supportful teaching exhibited a significant positive correlation with the total sports ethics orientation score (*r* = 0.304, *P* < 0.01) (Table 20). Perceptions of needs-supportive teaching exhibit significant positive correlations with dimensions of sports ethics orientation such as social norms, rule enforcement, and respect for opponents, while showing significant negative correlations with the instrumental aggression dimension. Furthermore, these perceptions demonstrate a significant positive correlation with the overall sports ethics orientation score. This indicates that the stronger high school students' perceptions of needs-supportive teaching, the higher their sports ethics levels tend to be, and the more likely they are to exhibit prosocial behavioral patterns.

**Table 20:** Correlation matrix of perceived teaching support and sports ethics orientation.

Subscale	M	SD	1	2	3	4	5	6
Demand-supported teaching perception	6.126	0.665	1					
Social norms	4.305	0.797	0.298^**^	1				
Rule enforcement	4.384	0.722	0.268^**^	0.783^**^	1			
Respect for opponents	4.135	0.875	0.287^**^	0.750^**^	0.689^**^	1		
Instrumental aggression	1.501	0.838	−0.222^**^	−0.752^**^	−0.664^**^	−0.670^**^	1	
Sportsmanship orientation	4.329	0.718	0.304^**^	0.932^**^	0.871^**^	0.878^**^	−0.869^**^	1

^**^At the 0.01 level (two-tailed), the correlation is significant.

H1: Physical education teachers‘ perceptions of supportive teaching can positively predict high school students' physical education ethical orientation.

#### 3.3.2 Perception of needs-supported instruction and physical education learning motivation

The total perceived support-oriented teaching score among high school students showed significant positive correlations with both intrinsic motivation (*r* = 0.464, *P* < 0.01) and identity regulation (*r* = 0.459, *P* < 0.01) within the physical education learning motivation dimension. Conversely, the total perceived need-supportive teaching score showed significant negative correlations with amotivation (*r* = −0.517, *P* < 0.01), external regulation (*r* = −0.467, *P* < 0.01), and introjected regulation (*r* = −0.563, *P* < 0.01). Furthermore, the total perceived demand-support teaching score showed a significant positive correlation with the total physical education learning motivation score (*r* = 0.559, *P* < 0.01) (Table 21). Perceived demand-supportive teaching exhibits a significant positive correlation with the autonomy-based motivation dimension (including intrinsic motivation and identity regulation) within physical education learning motivation, while showing a significant negative association with the control-based motivation dimension (encompassing amotivation, external regulation, and introjection). Furthermore, this perception showed a significant positive correlation with the total score of physical education learning motivation. This implies that the higher the perception of demand support from physical education teachers among high school students, the more conducive it is to the transformation of their physical education learning motivation toward autonomy.

**Table 21:** Correlation matrix of perceived demand-supportive teaching and motivation for physical education learning.

Subscale	M	SD	1	2	3	4	5	6	7
Need-supported teaching perception	6.126	0.665	1						
Unmotivated	1.796	0.935	−0.517^**^	1					
External regulation	1.965	1.032	−0.467^**^	0.710^**^	1				
Introverted regulation	2.047	1.19	−0.563^**^	0.565^**^	0.705^**^	1			
Identification regulation	5.75	1.111	0.459^**^	−0.681^**^	−0.818^**^	−0.694^**^	1		
Intrinsic motivation	5.912	1.092	0.464^**^	−0.706^**^	−0.802^**^	−0.642^**^	0.819^**^	1	
Physical education learning motivation	6.211	8.247	0.559^**^	−0.878^**^	−0.916^**^	−0.776^**^	0.888^**^	0.909^**^	1

^**^At the 0.01 level (two-tailed), the correlation is significant.

H2: Perceived supportiveness of physical education teachers can positively predict high school students' motivation to learn physical education.

#### 3.3.3 Motivation for physical education learning and ethical orientation in physical education

The total score of physical education learning motivation showed significant positive correlations with three factors under the dimension of ethical orientation in physical education: social norms (*r* = 0.359, *P* < 0.01), rule-based refereeing (*r* = 0.344, *P* < 0.01), and respect for opponents (*r* = 0.352, *P* < 0.01). Conversely, the total score of sports learning motivation showed a significant negative correlation with instrumental aggression (*r* = −0.300, *P* < 0.01). Furthermore, the total score of sports learning motivation also exhibited a significant positive correlation with the total score of sports ethical orientation (*r* = 0.382, *P* < 0.01) (Table 22). Sports learning motivation exhibits a significant positive correlation with dimensions such as social norms, rule enforcement, and respect for opponents within sports ethical orientation, while showing a significant negative association with the instrumental aggression dimension. Furthermore, this motivation also demonstrates a significant positive correlation with the overall score of sports ethical orientation. This indicates that sports learning motivation not only influences students' participation in physical education but also shapes their sports ethical orientation to a certain extent.

**Table 22:** Correlation matrix of sports learning motivation and sports moral orientation.

Subscale	M	SD	1	2	3	4	5	6
Motivation for physical education	6.211	8.247	1					
Social norms	4.305	0.797	0.359^**^	1				
Rule enforcement	4.384	0.722	0.344^**^	0.783^**^	1			
Respect for opponents	4.135	0.875	0.352^**^	0.750^**^	0.689^**^	1		
Instrumental aggression	1.501	0.838	−0.300^**^	−0.752^**^	−0.664^**^	−0.670^**^	1	
Ethical orientation in sports	4.329	0.718	0.382^**^	0.932^**^	0.871^**^	0.878^**^	−0.869^**^	1

^**^At the 0.01 level (two-tailed), the correlation is significant.

H3: The physical education learning motivation of high school students can positively predict their physical education ethical orientation.

### 3.4 Analysis of the mediating role of sports learning motivation between perceived supportiveness of instruction and sport ethics orientation

#### 3.4.1 Mediation

**Figure 1:**
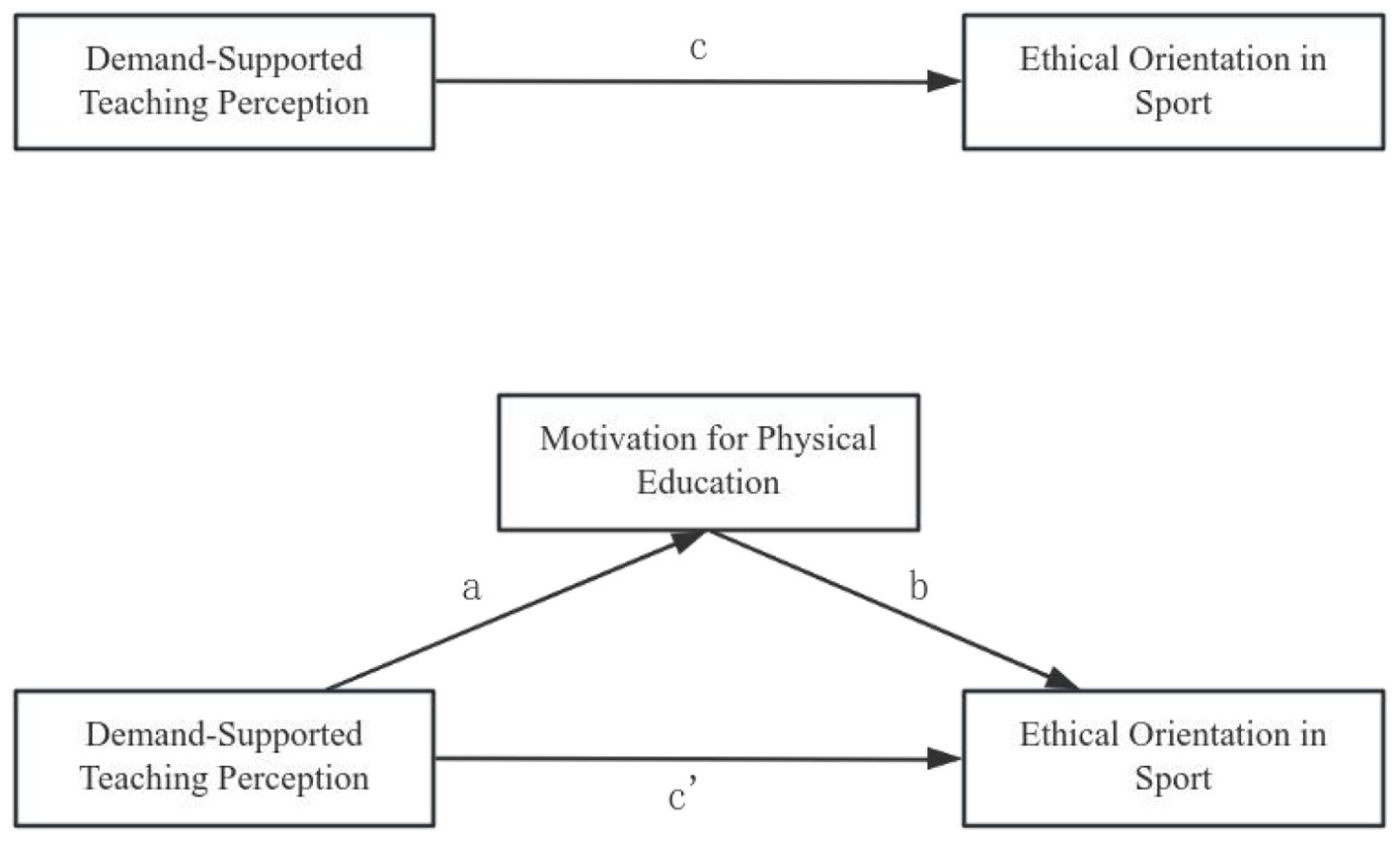
Mediation model path diagram. **(a, c)** represent the standardized regression coefficients for demand-supportive teaching perception when predicting physical education learning motivation and ethical orientation in isolation, respectively. **(b, c')** denote the respective standardized regression coefficients for physical education learning motivation and demand-supportive teaching perception when jointly predicting ethical orientation.

The above analysis demonstrates that the relationships among demand-support teaching perceptions, physical education learning motivation, and physical education ethical orientation satisfy the conditions for mediational testing. To investigate whether sports learning motivation mediates the relationship between perceived demand-supportive teaching and sports ethical orientation, this study conducted analyses following the mediation effect testing procedure and mediation model path diagram (Figure 1) proposed by (Wen et al. 2004). Using SPSS 27.0 software, we first conducted a three-step regression analysis, then constructed a mediation model, and finally utilized the PROCESS plugin to test the mediating effect.

First, conduct a three-step regression analysis:

Step 1: Perform regression analysis with perceived demand-supportive teaching as the predictor variable and sports ethics orientation as the dependent variable. The regression coefficient is 0.304, and the regression coefficient is significant (Table 23);

**Table 23:** The regression of perceived demand support teaching on sports moral orientation.

Dependent variable	Predictor variables	*R*	*R^2^*	Adjust *R^2^*	Beta	*t*	*P*
Ethical orientation in sports	Perceived demand-supported instruction	0.304	0.092	0.091	0.304	7.295^***^	0.000

^***^P < 0.001.

Step 2: Using demand-supported teaching perception as the predictor variable once again, but this time employing physical education learning motivation as the dependent variable, a regression analysis was conducted. The regression coefficient was 0.559, and the regression coefficient was significant (Table 24);

**Table 24:** The regression of demand support teaching perception on physical education learning motivation.

Dependent variable	Predictor variables	*R*	*R^2^*	Adjust *R^2^*	Beta	*t*	*P*
Motivation for physical education learning	Perceived demand-supported instruction	0.559	0.312	0.311	0.559	15.406^***^	0.000

^***^P < 0.001.

Step 3: Conduct regression analysis with perceived need-supportive teaching and physical education learning motivation as predictor variables, and physical education ethical orientation as the dependent variable. The regression coefficient for physical education learning motivation was 0.308, indicating statistical significance. This suggests that physical education learning motivation mediates the relationship between perceived need-supportive teaching and physical education ethical orientation. The regression coefficient for perceived demand-supportive teaching was 0.132, which was statistically significant. This indicates that physical education learning motivation partially mediates the relationship between perceived demand-supportive teaching and ethical orientation in physical education (Table 25).

**Table 25:** Regression of perceived demand-supportive teaching, physical education learning motivation on sports moral orientation.

Dependent variable	Predictor variable	*R*	*R^2^*	Adjust *R^2^*	Beta	*t*	*P*
Ethical orientation in sports	Demand-supported teaching perception	0.397	0.158	0.154	0.132	2.722^**^	0.007
	Motivation for physical education learning				0.308	6.357^***^	0.000

^**^P < 0.01.

^***^P < 0.001.

**Figure 2:**
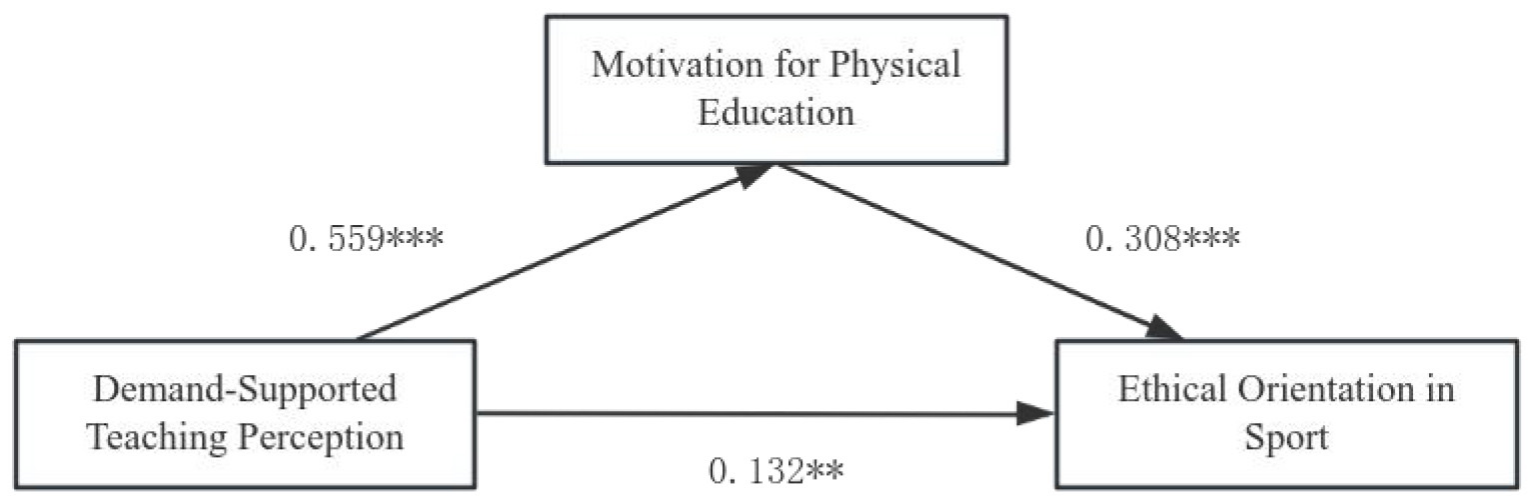
Mediation effect model diagram. ^**^P < 0.01. ^***^P < 0.001.

Second, based on the mediation effect and standardized regression equations (Table 26), construct a mediation model (Figure 2).

**Table 26:** The mediating effect of sports learning motivation.

Inspection procedure	Standardized regression equation	Regression coefficient test	
Step 1	Y = 0.304X	SE = 0.045	*t =* 7.295^***^
Step 2	M = 0.559X	SE = 0.450	*t =* 15.406^***^
Step 3	Y = 0.308M + 0.132X	SE = 0.052	*t =* 6.357^***^
		SE = 0.004	*t =* 2.722^**^

^**^P < 0.01.

^***^P < 0.001.

#### 3.4.2 Mediation effect test

To further validate the results of the mediation analysis, the following operations were conducted in SPSS software: “Perceived Demand-Supportive Instruction” was set as the independent variable, “Ethical Orientation in Physical Education” as the dependent variable, and “Motivation for Physical Education Learning” as the mediating variable. The Bootstrap method within the PROCESS plugin was employed to test the mediating effect. Table 27 shows that the total effect of “perceived need-supportive teaching” on “sports ethical orientation” is significant [effect size = 0.328, Boot SE = 0.045, Boot 95% CI (0.240, 0.417)]; Direct effect: After controlling for “sports learning motivation,” the direct effect of “perceived demand-supportive teaching” on “sports ethical orientation” was significant [effect size = 0.143, Boot SE = 0.052, Boot 95% CI (0.040, 0.245)], accounting for 43.4% of the total effect; Indirect effect: The mediating effect of “physical education learning motivation” was significant [effect size = 0.186, Boot SE = 0.038, Boot 95% CI (0.118, 0.267)], accounting for 56.6% of the total effect.

**Table 27:** Analysis of mediation effects.

Effect relationship	Effect value	Boot SE	Boot LLCI	Boot ULCI	Effect proportion
Total effect	0.328	0.045	0.240	0.417	
Direct effect	0.143	0.052	0.040	0.245	43.4%
Indirect effect	0.186	0.038	0.118	0.267	56.6%

Hypothesis H4: The mediating role of high school students' physical education learning motivation in the influence of perceived demand-supportive teaching by physical education teachers on their physical education ethical orientation is established.

## 4 Discussion

### 4.1 Analysis of the relationship between perception of needs-supportive teaching and sport ethics orientation

The results of this study indicate that high school students' perceived need support positively predicts their sports ethics orientation (*r* = 0.304, *P* < 0.01). Specifically, the sense of need support provided by physical education teachers not only has a positive impact on overall sports ethics orientation but also serves as a positive predictor of various sub-dimensions of sports ethics (such as social norms, rules and officiating, and respect for opponents), while simultaneously serving as a negative predictor of instrumental aggression.

This finding aligns with Self-Determination Theory, which holds that environments satisfying basic psychological needs promote positive behavioral development (Deci and Ryan, 2000). Empirical research confirms that psychological need satisfaction fosters prosocial behavior in adolescent student-athletes, while need frustration increases antisocial conduct (Sun et al., 2025). Longitudinal and multilevel studies further demonstrate that autonomy-supportive teaching enhances prosocial behavior and reduces antisocial behavior through basic need satisfaction (Cheon et al., 2023). Although direct investigations of need-supportive instruction and sport ethics orientation remain scarce, indirect evidence is compelling: Hui et al. found physical exercise positively predicts prosocial behavior via learning motivation, and prosocial behavior overlaps conceptually with sport ethics dimensions (e.g., respect for opponents, rule adherence) (Hui et al., 2022). Hoyo-Guillot et al. likewise showed supportive teaching elevates intrinsic motivation and prosocial climate perceptions (Hoyo-Guillot et al., 2025).

From an SDT perspective, need-supportive teaching—characterized by choice provision, positive feedback, and avoidance of coercion—satisfies autonomy, competence, and relatedness needs. Research with student-athletes indicates perceived autonomy support predicts both prosocial and antisocial moral behaviors, mediated by basic need satisfaction (Choi et al., 2026). Need satisfaction thus encourages internalization of moral norms rather than compliance driven by external contingencies. In sport settings, need-supportive coaching is associated with improved moral functioning, including reduced antisocial behavior toward opponents and referees (Delrue et al., 2017). Hence, a need-supportive teaching climate enables students to pursue fair competition and ethical conduct intrinsically, underscoring the value of supportive classroom environments for cultivating sport ethics.

### 4.2 Analysis of the relationship between perceived needs-supported instruction and physical education learning motivation

The results of this study indicate that high school students' perceived need support positively predicts their motivation to learn physical education (*r* = 0.559, *P* < 0.01). Specifically, the perceived need support provided by physical education teachers not only has a positive effect on overall motivation to learn physical education but also significantly and positively predicts forms of autonomous motivation, such as intrinsic motivation and identification regulation, while negatively predicting controlling or unmotivated states, such as amotivation, external regulation, and introjected regulation.

A systematic review of 105 studies confirms that need-supportive teaching strategies foster autonomous motivation and positive learning outcomes in physical education (Zhou et al., 2025). Longitudinal evidence further demonstrates that perceived need support positively predicts perceived usefulness and academic performance over time (Leo et al., 2025). Cross-sectional research with high school students shows that need support from physical education teachers promotes class engagement through basic psychological need satisfaction and intrinsic motivation (Do and Yoo, 2015). Multiple empirical studies consistently document that teacher autonomy, competence, and relatedness support enhance students' autonomous motivation (Maldonado et al., 2019; Guo et al., 2025; Su and Liu, 2025; Miao et al., 2024). RCT evidence provides causal confirmation: an SDT-based curriculum significantly boosted high school students' intrinsic motivation (d = 1.28), with need satisfaction explaining 87% of variance (Meng and Liu, 2026); a cluster RCT found need-supportive interventions enhanced autonomous motivation (β = 0.61) (Meerits et al., 2025). Pedagogically, need-supportive teaching operates through autonomy support (decision-making space), competence support (appropriately challenging tasks with feedback), and relatedness support (respectful teacher-student interactions). Students with high self-determination profiles exhibit greater intrinsic motivation and need satisfaction, whereas low self-determination profiles are linked to amotivation—underscoring the value of autonomy-supportive, inclusive climates for fostering physical education learning motivation (Gómez-López et al., 2025).

### 4.3 Analysis of the relationship between physical education learning motivation and ethical orientation in physical education

The results of this study indicate that high school students' motivation for physical education positively predicts their sports ethics orientation (*r* = 0.382, *P* < 0.01). Specifically, high-quality, autonomous motivation for physical education not only has a positive impact on overall sports ethics orientation but also positively predicts specific dimensions such as adherence to social norms and respect for rules, referees, and opponents, while negatively predicting instrumental aggressive behavior.

Grounded in self-determination theory, research consistently demonstrates that autonomous motivation is positively associated with adaptive outcomes and prosocial tendencies, whereas controlled motivation and amotivation are linked to maladaptive outcomes and antisocial conduct. A meta-analysis of 265 studies in school physical education confirmed a positive correlation between autonomous motivation and adaptive outcomes (Vasconcellos et al., 2020). Empirical evidence indicates that physical education learning motivation positively predicts prosocial behavior among middle school students (Hui et al., 2022), and that interventions designed to enhance intrinsic motivation effectively promote students‘ perception of a prosocial atmosphere (Hoyo-Guillot et al., 2025). Further, a study of 707 secondary school students identified four distinct motivational profiles, with the autonomous profile associated with significantly higher levels of sportsmanship across all dimensions—including respect for social conventions, respect for rules and teachers, full commitment, and respect for classmates—relative to controlled or amotivated profiles (Burgueño et al., 2020). Similarly, it has been reported that a coach-created mastery climate negatively predicts antisocial behavior (β = −0.27), whereas a performance climate positively predicts such conduct (β = 0.24), with moral disengagement acting as a key mediating and moderating mechanism (Zhong and Wang, 2025). Psychologically, when motivation stems from internal or deeply internalized sources, students focus on the intrinsic value of sport, enabling rational competition and greater respect for opponents and rules. Thus, cultivating high-quality motivation in physical education is essential not only for instructional effectiveness but also for fostering students' sports ethics and social development.

### 4.4 Analysis of the mediating effect of motivation in physical education learning

The results of this study indicate that the perception of demand-supportive instruction not only directly enhances students' ethical orientation toward physical education but also indirectly promotes its development by increasing their motivation to learn physical education (Effect Proportion = 56.6%).

Although studies directly testing the mediational mechanism among these three specific variables remain limited, extensive empirical research supports the core pathway of “motivation as a bridge between environment and behavior”: intrinsic motivation has been confirmed to mediate the link between teacher autonomy support and college students‘ sport participation (Guo et al., 2025), and physical education learning motivation similarly bridges teacher support and student sport participation (Su and Liu, 2025) as well as perceived diversity support and middle school students' learning engagement (Miao et al., 2024). Autonomous motivation has also been shown to mediate the relationship between multiple social supports and university students‘ academic engagement, with basic psychological need satisfaction and autonomous motivation acting as sequential mediators (Li et al., 2025). In physical education settings, teacher support directly influences learning motivation (effect size = 0.132) and indirectly via self-efficacy (effect size = 0.111) and chain mediation pathways (Hao et al., 2025). A 12-week need-supportive curriculum intervention significantly enhanced intrinsic motivation (d = 1.28), with need satisfaction explaining 87% of variance (Meng and Liu, 2026). Further, autonomous motivation mediates the association between perceived teacher autonomy support and adolescents' physical activity habits and reduced class non-participation (Jankauskiene et al., 2022).

Collectively, these findings underscore that need-supportive environments yield positive effects largely through intrinsic motivation activation. By extending this mediational model from academic engagement to ethical development, this study suggests that cultivating a need-supportive climate and high-quality learning motivation offers a more fundamental pathway to fostering sports ethics than behavioral discipline alone.

## 5 Strengths and limitations

Whilst this study has preliminarily validated the relationship between perceived demand-supportive teaching, physical education learning motivation, and ethical orientation in physical education, it remains subject to the following limitations: on the one hand, the cross-sectional design and self-report questionnaire methodology employed constrain the reliability of causal inferences and may introduce common method bias; on the other hand, the sample predominantly comprises senior secondary school students from Chongqing's urban districts, necessitating further validation of the findings' generalizability. Consequently, subsequent research may employ longitudinal tracking designs to deepen the validation of causal relationships between variables. Furthermore, integrating diverse methodologies such as experiments and interviews could broaden sample coverage and incorporate more varied research subjects. This approach would yield richer, more reliable primary data, thereby expanding both the depth and breadth of research on this topic.

## 6 Conclusion

This study examined the effects of perceived need-supportive instruction on Chinese high school students' physical education moral orientation, as well as the mediating role of physical education learning motivation. The results indicated that all three variables were significantly and positively correlated with one another. Specifically, perceived need-supportive instruction positively predicted both physical education moral orientation and physical education learning motivation, and physical education learning motivation also positively predicted physical education moral orientation. Furthermore, physical education learning motivation partially mediates the relationship between perceived need-supportive instruction and physical education moral orientation. This suggests that the more autonomy, competence, and emotional support students perceive from their teachers, the stronger their physical education learning motivation becomes, which in turn contributes to the formation of a more positive physical education moral orientation. Need-supportive instruction not only directly enhances students' physical education moral orientation but also indirectly promotes its development by strengthening learning motivation.

## Data Availability

The original contributions presented in the study are included in the article/Supplementary material, further inquiries can be directed to the corresponding author.
