# Supplementary material for: The effect of perceived supportiveness of instruction on high school students' sport ethics orientation: the mediating role of sport learning motivation
Source: Front Psychol. 2026 Jun 5;17:1790018. doi: 10.3389/fpsyg.2026.1790018 (PMC13279086; doi:10.3389/fpsyg.2026.1790018)
Supplement: Supplementary file 1 [file Supplementary_File_1.docx]

Survey Questionnaire on the Impact of High School Students’ Perceived Support Needs on Their Attitudes Toward Sports Ethics

Dear Students:

Hello!

To gain a deeper understanding of your perceptions of need-supportive teaching in physical education classes, your levels of motivation for physical education, and your attitudes toward sports ethics—and thereby enhance your learning experience and outcomes in these classes—we are conducting this survey. Please carefully complete the “Physical Education Need-Supportive Teaching Perception Scale,” the “Physical Education Learning Motivation Scale,” and the “Multidimensional Sports Ethics Orientation Scale.” All information collected through this survey is intended solely for academic research purposes. We will strictly protect your personal information and ensure that it will not affect your daily life in any way. There are no standard answers to any of the questions in the questionnaire, and only one option may be selected per question. Your honest feedback is crucial to us. We sincerely appreciate your strong support and active cooperation!

**Part 1: Basic Information**

Gender： □Male □Female

Grade： □10th grade □11th grade □12th grade

Are you an only child? □Yes □No

**Part 2: Perception Scale of Supportive Teaching in Physical Education Classes**

This scale has seven levels, with each of the seven options representing a different meaning (1 = Strongly Disagree, 2 = Disagree, 3 = Somewhat Disagree, 4 = Not Sure, 5 = Somewhat Agree, 6 = Agree, 7 = Strongly Agree). Please mark the appropriate option based on your actual situation“√”.

| Essay Questions | Does not apply at all | Does not apply | Applies to some extent | Not sure | Applies to some extent | Applies | Applies very much |
| --- | --- | --- | --- | --- | --- | --- | --- |
| 1. Our PE teacher gives us plenty of opportunities to choose during class. | □ | □ | □ | □ | □ | □ | □ |
| 2. The PE teacher makes us feel capable of completing the activities in class. | □ | □ | □ | □ | □ | □ | □ |
| 3. Our PE teacher treats us with great respect. | □ | □ | □ | □ | □ | □ | □ |
| 4. Our PE teacher often encourages us to ask questions in class. | □ | □ | □ | □ | □ | □ | □ |
| 5. Our PE teacher makes us feel like we’re good at sports. | □ | □ | □ | □ | □ | □ | □ |
| 6. Our PE teacher cares deeply about us. | □ | □ | □ | □ | □ | □ | □ |
| 7. During class, our PE teacher listens carefully to our ideas about what we plan to do. | □ | □ | □ | □ | □ | □ | □ |
| 8. Our PE teacher helps us improve in our physical education studies. | □ | □ | □ | □ | □ | □ | □ |
| 9. Our PE teacher is very friendly toward us. | □ | □ | □ | □ | □ | □ | □ |
| 10. Before suggesting a new solution, our PE teacher makes an effort to understand our thoughts. | □ | □ | □ | □ | □ | □ | □ |
| 11. Our PE teacher makes us feel like we can do better in PE class. | □ | □ | □ | □ | □ | □ | □ |

**Part 3: Physical Education Motivation Scale**

This scale has seven levels, with each of the seven options representing a different meaning (1 = Strongly Disagree, 2 = Disagree, 3 = Somewhat Disagree, 4 = Not Sure, 5 = Somewhat Agree, 6 = Agree, 7 = Strongly Agree). Please mark the appropriate option based on your actual situation“√”.

| Essay Questions | Does not apply at all | Does not apply | Applies to some extent | Not sure | Applies to some extent | Applies | Applies very much |
| --- | --- | --- | --- | --- | --- | --- | --- |
| 1. I really don’t know why I have to take PE class. | □ | □ | □ | □ | □ | □ | □ |
| 2. I take PE class to avoid getting a bad grade. | □ | □ | □ | □ | □ | □ | □ |
| 3. If I didn’t take PE class, I’d feel terrible. | □ | □ | □ | □ | □ | □ | □ |
| 4. I take PE because making progress on the assignments is important to me. | □ | □ | □ | □ | □ | □ | □ |
| 5. I take PE because it’s enjoyable. | □ | □ | □ | □ | □ | □ | □ |
| 6. I don’t know why we have to take PE. | □ | □ | □ | □ | □ | □ | □ |
| 7. I take PE to avoid criticism from the PE teacher. | □ | □ | □ | □ | □ | □ | □ |
| 8. If I couldn’t take PE, I’d feel uneasy. | □ | □ | □ | □ | □ | □ | □ |
| 9. I take PE because mastering and excelling at the sports taught in class is important to me. | □ | □ | □ | □ | □ | □ | □ |
| 10. I take PE because it’s exciting. | □ | □ | □ | □ | □ | □ | □ |
| 11. I really feel like taking PE is a waste of my time. | □ | □ | □ | □ | □ | □ | □ |
| 12. I take PE because it’s a required class at school. | □ | □ | □ | □ | □ | □ | □ |
| 13. I take PE because it’s important for me to keep trying my best in class. | □ | □ | □ | □ | □ | □ | □ |
| 14. I take PE because it’s fun. | □ | □ | □ | □ | □ | □ | □ |
| 15. I don’t know what I’ve learned from PE. | □ | □ | □ | □ | □ | □ | □ |

**Part 4: The Multidimensional Sports Ethics Orientation Scale**

This scale has five levels, with each of the five options representing a different meaning (1 = Strongly Disagree, 2 = Disagree, 3 = Not Sure, 4 = Agree, 5 = Strongly Agree). Please mark the appropriate option based on your actual situation“√”.

| Essay Questions | Does not apply at all | Does not apply much | Not sure | Applies somewhat | Applies completely |
| --- | --- | --- | --- | --- | --- |
| 1. Even if I lose, I will congratulate my opponent, no matter who they are. | □ | □ | □ | □ | □ |
| 2. I can accept the referee’s decisions. | □ | □ | □ | □ | □ |
| 3. If my opponent unexpectedly falls during a match, I will help them get back up. | □ | □ | □ | □ | □ |
| 4. To win a match, I often resort to verbal abuse or intimidation to distract my opponent. | □ | □ | □ | □ | □ |
| 5. Even if I lose a match, I will still shake hands with my opponent’s coach. | □ | □ | □ | □ | □ |
| 6. I can follow the rules of the competition. | □ | □ | □ | □ | □ |
| 7. Under appropriate circumstances, I will ask the referee to allow an opponent disqualified due to a misjudgment to continue competing. | □ | □ | □ | □ | □ |
| 8. I often use provocative behavior to prevent my opponent from winning. | □ | □ | □ | □ | □ |
| 9. After winning a match, I still acknowledge my opponent’s outstanding performance. | □ | □ | □ | □ | □ |
| 10. I can genuinely abide by all the rules of the sport I participate in. | □ | □ | □ | □ | □ |
| 11. If my opponent is accidentally injured, I will ask the referee to stop the match promptly and help my opponent treat the injury. | □ | □ | □ | □ | □ |
| 12. I often resort to any means necessary against strong opponents to intimidate them. | □ | □ | □ | □ | □ |
| 13. Regardless of the outcome, I always shake hands with my opponent after the match. | □ | □ | □ | □ | □ |
| 14. Even if the referee is not a physical education teacher, I respect his or her decisions. | □ | □ | □ | □ | □ |
| 15. If I see an opponent being penalized due to a misjudgment, I will try to rectify the situation. | □ | □ | □ | □ | □ |
| 16. I often use brute force to pressure my opponent, hoping they will make a mistake. | □ | □ | □ | □ | □ |
| 17. After the match, I will congratulate my opponent on their outstanding performance. | □ | □ | □ | □ | □ |

Scoring Methods for Each Subscale

Scoring Method for the Physical Education Class Support-Oriented Teaching Perception Scale

Subscales:

Autonomous Support: 1, 4, 7, 10

Competence Support: 2, 5, 8, 11

Emotional Support: 3, 6, 9

Scoring Method for the Physical Education Class Learning Motivation Scale

Subscales:

Amotivation: 1, 6, 11, 15

External Regulation: 2, 7, 12

Introjected Regulation: 3, 8

Identification Regulation: 4, 9, 13

Internal Regulation: 5, 10, 14

Physical Education Learning Motivation Formula: Internal Regulation × 2 + Identification Regulation – Introjected Regulation – External Regulation × 2 – Unmotivated × 3

Multidimensional Physical Education Ethical Orientation Scale Scoring Method

Subscales:

Social Norms: 1, 5, 9, 13, 17

Rules and Referees: 2, 6, 10, 14

Respect for Opponents: 3, 7, 11, 15

Instrumental Aggression: 4, 8, 12, 16
